# Conditions Mimicking the Cancer Microenvironment Modulate the Functional Outcome of Human Chorionic Villus Mesenchymal Stem/Stromal Cells *in vitro*

**DOI:** 10.3389/fcell.2021.650125

**Published:** 2021-06-21

**Authors:** Yasser Basmaeil, Abdullah Al Subayyil, Mohammad Abumaree, Tanvir Khatlani

**Affiliations:** Stem Cells and Regenerative Medicine Department, King Abdullah International Medical Research Center, King Saud bin Abdulal Aziz University for Health Sciences, King Abdulaziz Medical City, Ministry of National Guard Health Affairs, Riyadh, Saudi Arabia

**Keywords:** placenta, chorionic villous MSCs, MDA231, conditioned medium, adhesion, proliferation, migration, invasion

## Abstract

Mesenchymal stem/stromal cells isolated from chorionic villi of human term placentae (CV-MSCs) possess unique biological characters. They exhibit self-renewal, directional migration, differentiation, and immunomodulatory effects on other cell lineages, by virtue of which they can be utilized as therapeutic carriers, for drug targeting, and therapy. Tumors display characteristic features of a damaged tissue microenvironment, which is saturated with conditions such as hypoxia, sustained inflammation, and increased oxidative stress. CV-MSCs function normally in a high oxidative stress environment induced by hydrogen peroxide (H_2_O_2_) and glucose and also protect endothelial cells from their damaging effects. For their therapeutic applications in a disease like cancer, it is necessary to ascertain the effects of tumor microenvironment on their functional outcome. In this study, we investigated the functional activities, of CV-MSCs in response to conditioned media (CM) obtained from the culture of breast cancer cell line MDA-231 (CM-MDA231). CV-MSCs were exposed to CM-MDA231 for different spatio-temporal conditions, and their biological functions as well as modulation in gene expression were evaluated. Effect of CM-MDA231 on factors responsible for changes in functional outcome were also investigated at the protein levels. CV-MSCs exhibited significant reduction in proliferation but increased adhesion and migration after CM-MDA231 treatment. Interestingly, there was no change in their invasion potential. CM-MDA231 treatment modulated expression of various genes involved in important cellular events including, integration, survival, message delivery and favorable outcome after transplantation. Analysis of pathways related to cell cycle regulation revealed significant changes in the expression of p53, and increased phosphorylation of Retinoblastoma (Rb) and Checkpoint Kinase 2 in CV-MSCs treated with CM-MDA231. To summarize, these data reveal that CV-MSCs retain the ability to survive, adhere, and migrate after sustained treatment with CM-MDA231, a medium that mimics the cancer microenvironment. These properties of CV-MSCs to withstand the inflammatory tumor like microenvironment prove that they may make useful candidate in a stem cell based therapy against cancer. However, further pre-clinical studies are needed to validate their therapeutic usage.

## Introduction

Cancer is the second leading cause of death globally after cardiovascular disease. An estimated count of 9.6 million people died because of cancer in 2018, as per the latest WHO report. It accounts for about 1 in 6 deaths globally (Cancer, 2020; A WHO Report). In spite of many recent breakthroughs in the research on diagnostic and treatment options against cancer, it still poses a threat to humanity. In order to design better therapeutic modules, comprehensive information need to be acquired in the biology of this disease (Chu et al., 2020). Although, many treatment options including surgery, radiotherapy, chemotherapy, cancer vaccines, immunotherapy and CAR-T-cell therapy are now available and used successfully against many cancers, yet the overall outcome is abysmal because of multiple reasons. It may because of the non-specific targets, off target effects, therapy resistance and tumor recurrence, etc. Based on these facts, newer therapeutic methods need to be explored and tested. Various cell-based therapies are being investigated in pre-clinical trials as they show great promise in the fight against cancer. Among them, stem cell therapy (whereby stem cells are used as therapeutic agents) has provided an excellent alternative option. Stem cells are preferred for their therapeutic efficacy because of their enhanced targeting of tumors and minimizing the off-target effects (Gomes et al., 2017).

Mesenchymal stem cells (MSCs) are undifferentiated stromal cells which are adherent in nature and are localized in different tissues and organs such as bone marrow, adipose tissue, dental pulp, umbilical cord and placenta (Nwabo Kamdje et al., 2017). They differentiate into multiple cell lineages that include the chondrocytes, astrocytes, and adipocytes. According to the International Society for Stem Cell Research (ISSCR) guidelines, adult mesenchymal cells must express CD90, CD73, and CD 105, and must be lacking the expression of CD45, CD34, CD31, CD14, CD19, and HLA-DR, to qualify for being called as stem cells (Oswald et al., 2004; Phinney and Sensebé, 2013). Multiple characteristics of MSC’s such as immunosuppression, positive role in angiogenesis and their proliferative and migratory potential make them suitable agents in regenerative medicine as well as for their therapeutic role in various inflammatory diseases.

Previously, we have reported the isolation and characterization of MSCs from the Chorionic Villi of human term placentae (CV-MSCs) (Abumaree et al., 2013a). CV-MSCs exhibit self-renewal properties. They differentiate into a three cell lineages including adipocytes, osteocytes and chondrocytes, which is the hallmark of stemness of MSCs (Abumaree et al., 2013b). CV-MSCs stimulate the generation of immunosuppressive M2 macrophages from human monocytes (Al Jumah and Abumaree, 2012; Abumaree et al., 2013a,b). In addition, they also modulate the functions of T-lymphocytes and dendritic cells (Abomaray et al., 2015). These immunosuppressive properties of CV-MSCs make them an attractive source for allogeneic transplantation while reducing the risk of rejection by patient immune system, thereby increasing their success in the rate of transplantation. Furthermore, CV-MSCs not only function normally in a high oxidative stress environment induced by Hydrogen Peroxide (H_2_O_2_) and glucose (Abumaree et al., 2017; Basmaeil et al., 2018), but also protect the endothelial cells from their harmful effects (Abumaree et al., 2017; Basmaeil et al., 2018). They did not inhibit the cytolytic activity of natural killer (NK), but in turn induced expression of a variety of anti-tumor molecules by NK cells (Abumaree et al., 2019). Collectively, these functional characteristics of CV-MSCs make them a promising therapeutic tool in regenerative medicine and antitumor therapy.

Although multiple studies have demonstrated encouraging response of MSCs toward the therapeutic potential for inflammatory diseases, yet the cells still face challenge of hostile microenvironment in disease like cancer, that largely limit their functional outcome (Murakami et al., 2015; Kil et al., 2016; Fu et al., 2017; Lee and Hong, 2017; Liang et al., 2017; Mohr and Zwacka, 2018). Tumors, which behave like wounded tissues, possess the characteristic features of a wound in their microenvironment, which include events such as hypoxia, mechanical stress, sustained inflammation and oxidative stress (Wang et al., 2009; Pattabiraman and Weinberg, 2014). The microenvironment of developing tumor constitutes actively dividing tumor cells, the tumor stromal cells, developing blood vessels, immune cells, inflammatory and other associated tissue cells. This unique environment emerges in the course of tumor progression in association with the host tissues. Since tumor is the dominating factor, this microenvironment shapes the molecular and cellular events happening in the niche (Whiteside, 2008). Because of these cellular events, the microenvironment gets saturated with a plethora of inflammatory molecules including cytokines, chemokines, growth factors, and other inflammatory effectors, which although are favorable for tumor growth and metastasis, yet they might influence negatively on the anti-tumor properties of the MSCs (Alphonso and Alahari, 2009; Uchibori et al., 2009; Burr et al., 2013; English, 2013; Yang et al., 2013; Arnold et al., 2015; Guan, 2015; Lee et al., 2015; Murakami et al., 2015; Lee and Hong, 2017).

Based on the previous results, and in order to utilize CV-MSCs for successful anti-tumor therapies, it is essential to evaluate the consequential effects of cancer microenvironment on their functional outcome.

In this study, we have examined the effects of medium mimicking the cancer microenvironment on the functional outcome of CV-MSCs. We performed spatial and temporal treatment of CV-MSCs to the “Conditioned Medium” obtained from the culture of breast cancer cell line MDA-231 (CM-MDA231). We evaluated their functional and phenotypic properties including, proliferation, adhesion, migration and invasion. We evaluated the modulation in expression of important genes, which play significant roles in these functional outcomes. Finally, we analyzed the proteins related to cell cycle regulation in CV-MSCs treated with CM-MDA231 at the expression and activation levels.

## Materials and Methods

### Ethical Approval and Tissue Collection

The Institutional Review Board (IRB) of King Abdullah International Medical Research Centre (KAIMRC) approved this study vide the proposal number RC20/346/R. Human placentae and umbilical cord tissues from uncomplicated and healthy pregnancies delivered normally through vagina (38–40 weeks of gestation) were collected within 2 h of delivery. Written consent was obtained from the patients prior to the placenta collection. The gestational age and fetal viability were confirmed by ultrasound examinations from time to time during the gestational period. All procedures (clinical and experimental) were performed as per the research guidelines and regulations of KAIMRC.

### Isolation and Culture of CV-MSCs

CV-MSCs were isolated as previously described using our published explant method (Abumaree et al., 2013b). Briefly, about 40 mg of the chorionic villi tissue was washed with sterile PBS and incubated for overnight at 4°C in DMEM-F12 medium (Life Technologies, NY, United States) containing 2.5% trypsin (Life Technologies, NY, United States), 270 units/ml DNase (Life Technologies, NY, United States), and antibiotics (100 U/l penicillin and 100 μg/ml streptomycin). The tissue was further washed with PBS and placed in complete DMEM-F12 culture medium containing 10% MSC Certified Fetal Bovine Serum (Life Technologies, NY, United States), 100 μg/ml of L-glutamate, and antibiotics. Tissues were incubated at 37°C in a cell culture incubator containing 5% CO_2_. The migrated cells from the explants were harvested with TrypLE^TM^ Express detachment solution (Life Technologies, NY, United States) and characterized before being used for experimental purposes. At 75% confluency, the cells were harvested using TrypLE^®^ express detachment solution (Life Technologies, NY, United States), and characterized by flow cytometry, as already described (Abumaree et al., 2013b). CV-MSCs were prepared independently from five different placentae and were used at passages 3–4.

### Antibodies and Reagents

GAPDH and a panel of fluorescent-labeled antibodies (VCAM (cat#FAB5649P), ICAM (cat#BBA20), PECAM (cat#FAB3567P), E-Cadherin (cat#FAB18381P), Integrin α5 (cat#FAB1864P), Integrin-M (cat#FAB16991P), and EpCAM (cat#FAB9601P), used for flow cytometry were purchased from R&D Systems, MN, United States. p53 (cat#2527), pRb (S807) (cat#8516), pChk2 (T68) (cat#2661) and β-Actin (cat#3700 and cat#8457) antibodies for immunoblotting were purchased from Cell Signaling Technologies, MA, United States. Goat anti-rabbit and goat anti-mouse secondary antibodies (cat#21537 and 21538M) were purchased from Sigma-Aldrich, MO, United States. Protease inhibitor cocktail (cat#P8340) was from Millipore Sigma (Burlington, MA, United States).

### CM-MDA231 Collection and CV-MSC Treatment

Conditioned medium (CM) from the culture of breast cancer cell line, MDA-231 (MDA-MB-231, ATCC^®^ HTB-26^TM^, ATCC, Manassas, VA, United States) was produced using our previously published method (Abumaree et al., 2013b). Briefly, 1 × 10^5^ MDA-231 were cultured in DMEM-F12 culture medium with 10% FBS, 100 μg/ml L-glutamate and antibiotics (100 U/l penicillin and 100 μg/ml streptomycin) until cells reached 75% confluency. Cells were washed with PBS to remove any dead cells, then fed with fresh complete medium and incubated for further 72 h when conditioned medium (CM-MDA231) was collected, centrifuged to remove any dead cells, and stored at -80°C for future use.

For dose response and time course evaluation, the outcome was first measured while culturing the cells in regular complete medium (untreated control), or treating them with different dilutions of CM-MDA231 (1, 10, and 25%) and culturing for 24, 48, and 72 h, prior to analysis. For preconditioning experiments hereby called as “pre-treatment groups,” the cells were initially treated and incubated with CM-MDA231 for 24, 48, or 72 h. After treatment the cells were washed, fed with fresh medium (without CM-MDA231), and incubated for another 24 h, before assessing the functional outcome. For the comparison, the outcome of CV-MSCs was assessed simultaneously while incubating the cells with CM-MDA231 hereby called as “in-treatment group.”

Based on the treatment options, four treatment groups were designed to assess the dose response and time course for CM-MDA231 to show a response on CV-MSCs. They included untreated control, and three doses of CM-MDA231 (1, 10, and 25%) for dose response evaluation; and an untreated control, in-treatment group, and two pre-treated groups, for time course standardization after preconditioning.

### Analysis of Cellular Proliferation Using a Tetrazolium Compound (MTS Assay)

The treated and untreated control cells were seeded at a density of 5 × 10^3^ in 96-well tissue culture plates and incubated for 24 h at 37°C in a humidified cell culture incubator. MTS kit (CellTiter 96^®^ Aqueous Non-Radioactive Cell Proliferation Assay, cat#G5421, Promega, Germany) was used to assess the cellular proliferation, as instructed by the manufacturer. Briefly, 20 μl of MTS solution was added to each well containing 100 μl of growth medium, and incubated at 37°C in a cell culture incubator for 4 h. The absorbance was recorded at 490 nm using an ELISA plate reader (Spectra MR, Dynex Technologies, Denkendorf, Germany). Medium containing MTS solution without any cells was used as blank. The results were presented as means (± SD) obtained from three independent experiments. Cells from passages 3–5 were used in each experiment prepared from different placentae.

### Real-Time Cell Analyses (RTCA) Assay for Cellular Functions

Adhesion, proliferation and migration of treated and untreated cells were examined using the xCELLigence Real-Time Cell Analyzer (RTCA-DP version; Roche Diagnostics, Mannheim, Germany). This system monitors continuous cellular events by recording label-free changes in electrical impedance (reported as cell index) as already described (Limame et al., 2012). For adhesion and proliferation, we used “E-Plate 16” (cat#05469813001, Roche Diagnostics, IN, United States). Hundred microliter medium (with or without CM-MDA231) was added to each well of the plate, and background impedance was achieved as previously described (Abumaree et al., 2019). Each group of cells (treated and untreated control) were seeded in four wells and the plate was incubated at room temperature for 30 min to reach an equilibrium before putting it in the xCELLigence system at 37°C in a cell culture incubator. The cell index was monitored for 72 h. Cellular adhesion was measured after 2 h, and the rate of cell proliferation was calculated after 72 h. Data were analyzed using RTCA xCELLigence software (version 1.2.1) and final data for proliferation was demonstrated after normalizing it with the adhesion data. Data for proliferation is expressed as normalized cell index with mean and standard errors.

16-well plates, “CIM-16” (cat#05665825001, Roche Diagnostics, IN, United States) specially designed to record migrating cells across the chambers were used to record the migration potential of treated and untreated cells. The CIM-16 migration plates have upper and lower chambers separated by a porous (pore size 8 μm) polyethylene terephthalate (PET) membrane in conjunction with microelectrodes (Abumaree et al., 2019). Fifty microliter pre-warmed media was added to wells of the upper chamber, and the plates were locked in the RTCA device and incubated at 37°C in a cell culture incubator for 1 h to obtain equilibrium. Untreated or treated cells were seeded at a density of 20 × 10^3^ in the upper chamber in 100 μl medium and the plates were incubated at room temperature for 30 min to allow the cells to adhere to the membrane. Complete medium supplemented with 20% FBS was added to the lower chamber as a chemo-attractant for the migrating cells. The impedance value of each well was captured by the xCELLigence system after every 15 min for 24 h as described above. Experiments were performed with four independent samples and migration of cells observed in presence or absence of 20% FBS served as positive and negative controls, respectively.

### Transwell Assay for Cellular Migration and Invasion

We investigated the migratory potential and invasiveness of the CV-MSCs before and after treatment by performing the transwell assay. The cells were made to pass through 8-mm pore polycarbonate transwell inserts (for migration), and through the insert coated with Matrigel (cat#356235, BD Biosciences, San Jose, CA, United States) for determining cell invasion. Treated and untreated cells at the concentration of 2.5 × 10^3^ cells/ml were seeded with serum-free DMEM-F12 medium in the upper chamber of the insert. DMEM-F12 with 20% FBS was used as a chemo-attractant and added to the lower chamber of the plate. The cells were incubated for 48 h in a humidified cell incubator in 5% CO_2_ at 37°C. The invaded/migrated cells that had crossed the filter to the bottom chamber were washed with PBS, and fixed with 4% paraformaldehyde for 15 min at room temperature. Staining was done with 0.1% crystal violet stain and the cells were visualized and photographed under a light microscope (250X magnification). The migration and invasion rate was determined after counting the cells.

### Flow Cytometry

Treated and untreated cells were harvested as described earlier and 1 × 10^5^ of cells were stained using fluorescent monoclonal antibodies against the specific adhesion molecule as described above in “antibodies and reagents” section, and as described earlier (Basmaeil et al., 2020). Briefly, the cells were incubated with respective antibodies for 30 min and washed with cold PBS at 8°C. For analysis of intracellular expression of the proteins, the cells were fixed with 4% paraformaldehyde in sterile PBS at pH 7.4 for 10 min at room temperature, and permeabilized for 5 min at room temperature in 0.1% Saponin containing PBS. Intracellular and cell-surface protein expression was assayed by BD FACS CANTO II (Becton Dickinson, NJ, United States) flow cytometer. Cells treated with FITC or PE-labeled mouse IgG or IgM antibody were used as a negative control.

### RNA Isolation and Real-Time PCR Analysis (RT-PCR)

We used RNEasy mini kit (cat#74104, Qiagen, MD, United States) to isolate total RNA from treated and untreated cells. Fastlane cDNA Analysis Kit (cat#215011, Qiagen, MD, United States) was used to transcribe RNA into the single-stranded cDNA. Real-time PCR reaction was performed to detect the expression of 84 genes related to Human Cytokines & Chemokines using a RT^2^ Profiler Kit (cat#PAHS-150Z, Qiagen, Hilden, Germany) on the CFX96 real-time PCR detection system (Bio-Rad, CA, United States). Data were initially analyzed using the CFX manager software (Bio-Rad, CA, United States) and for further analyses were exported to Microsoft Excel. The data were analyzed by calculating ΔΔ^–2^ values, and expressed as fold change expression as compared to the relative expression of house-keeping genes used as a loading control. Experiments were performed in triplicate and repeated three times using CV-MSCs prepared from three independent placentae.

### Immunoblotting

After washing the treated and untreated cells with PBS to remove the dead cells and debris, 100 μl of cell lysis buffer (cat#9803, Cell Signaling Technologies, MA, United States) with protease and phosphatase inhibitors was added to each well. Cells were scraped and the lysate was collected, centrifuged at 15,000 rpm for 5 min at 4°C and stored at -80°C. Protein concentration was estimated by Bradford assay method before processing. 30 μg of extracted proteins with an equal amount (v/v) of 2X Laemmli sample buffer (cat#1610737, Bio-Rad, CA, United States) was boiled for 10 min, and loaded onto a 10% SDS-PAGE gel. Separated proteins were transferred onto a PVDF membrane using Mini Trans blot system (Bio-Rad, CA, United States) at 100 V for 120 min. The membranes were blocked in Tris-buffered saline with 0.1% (v/v) Tween 20 (TBS-T) containing 5% non-fat dry milk for 30 min at RT. Antibodies at 1:1,000 dilution in TBST buffer containing 5% skimmed milk or BSA, were added to the membranes and incubated overnight at 4°C. The membranes were washed three times with TBS-T, and incubated with horseradish peroxidase (HRP)-conjugated anti-goat or anti-rabbit secondary antibodies (R&D Systems, MN, United States) at 1:3,000 dilution for 2 h at room temperature. After washing three times with TBS-T, specific protein bands were visualized using SuperSignal^TM^ West Pico (cat#34577) or West Femto Chemiluminescent Substrate (cat#34096, Thermo Fisher Scientific, MA, United States) in a ChemiDoc visualization system (Bio-Rad, CA, United States). We used image-analyzing software Image Lab (Bio-Rad, CA, United States) for measuring the density of the bands, which were normalized with the bands obtained for β-Actin. Each experiment was performed three times using cells from passages three.

### Statistical Analysis

All the data shown in the bar graphs are presented as means ± standard deviation (SD) of three independent experiments. Each experiment was performed three times to avoid the bias. The data comparison was carried out between two groups by unpaired *t*−test. Data of single factor between two groups were compared by one−way analysis of variance (ANOVA), while data of double factors in multiple groups were compared by two−way ANOVA and a *p* ≤ 0.05 was considered to be statistically significant.

## Results

### Dose Response and Time Course Curve for Experimental Standardization

**FIGURE 1:**
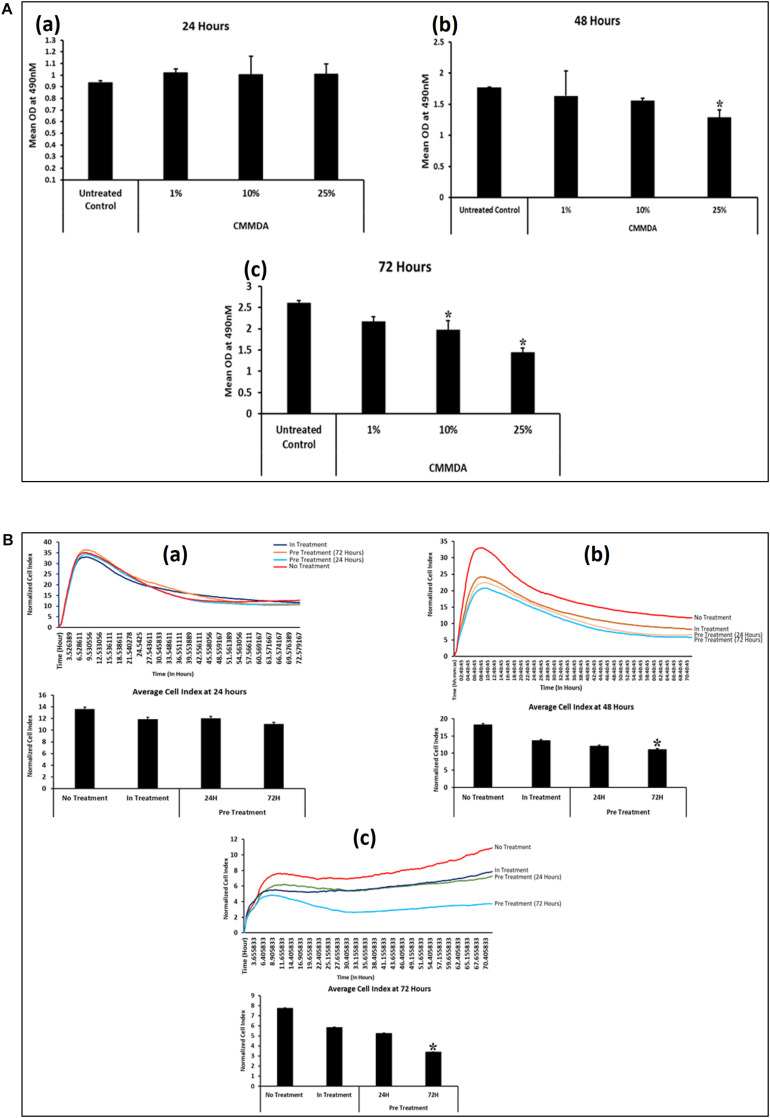
Time and CM-MDA231 dose curve for treatment of CV-MSCs. The effect of CV-MSCs in response to various concentrations (1–25%) of CM-MDA231 at specific time points (24–72 h) using MTS **(A)** and xCELLigence system **(B)**. At 24 h, CV-MSCs proliferation did not change in response to 1–25% CM-MDA231 as compared to untreated controls **(Aa)**. At 48 h, CV-MSCs proliferation did not change in response to 1 and 10% concentration of CM-MDA231, but reduced significantly in response to 25% CM-MDA231 **(Ab)**. At 72 h and as compared to untreated controls, CV-MSCs proliferation reduced significantly in response to 10 and 25% CM-MDA231, but did not change significantly in response to 1% CM-MDA231 and untreated controls **(Ac)**. CV-MSCs were preconditioned with CM-MDA231 for 24 and 72 h, cultured in complete medium for 24 h post preconditioning and then their functionalities were recorded in xCELLigence system **(B)**. CV-MSCs did not respond to preconditioning at 24 h as compared to in-treatment and untreated control **(Ba)**. However, significant changes were observed at 48 and 72 h, after preconditioning the CV-MSCs for 72 h as compared to in-treatment and untreated control **(Bb,Bc)**. Bars represent standard errors *P < 0.05.

To determine the appropriate dose of CM-MDA231, which has a measurable effect on the performance of CV-MSCs, we have initially selected three doses at 1, 10, and 25% of CM-MDA231. The cells were incubated for 24, 48, and 72 h and then MTS assay was performed. As shown in Figure 1A, the proliferation of CV-MSCs did not change after 24 h, in any of the treatment options as compared to the untreated control (Figure 1Aa). However, after 48 h of sustained treatment with CM-MDA231, the cells showed a dose dependent reduction in overall proliferation, which reached to the significant level (*p* < 0.05) at 25% CM-MDA231 against 1%, 10% and untreated control (Figure 1Ab). This response was more robust after 72 h of treatment, when significant decrease in proliferation at both 10 and 25% was observed, as compared to 1% treatment or untreated control (Figure 1Ac).

Effect of preconditioning of CV-MSCs with CM-MDA231 was determined using xCELLigence Real-Time Cell Analyzer system. After sustained incubation of cells, for 24 and 72 h with 25% CM-MDA231, the cells were washed with PBS, and fed with complete medium for 24, 48, and 72 h, before harvesting and subjecting to cell analysis by xCELLigence. Untreated cells in 25% CM-MDA231 (In treatment) and in complete medium served as an untreated control. Pre-conditioned cells did not show any change after 24 h post preconditioning in their functional outcome as compared to in treatment and untreated controls (Figure 1Ba). We found significant reduction (*p* < 0.05) in proliferation of CV-MSCs preconditioned for 72 h in 25% CM-MDA231 and cultured for further 48 h before measuring their functional outcome. This change was not significant for the cells preconditioned for 24 h, as compared to in-treatment and untreated control (Figure 1Bb). For the cells cultured for 72 h after preconditioning, there was no significant change in functional outcome as compared to 48 h post preconditioning (Figure 1Bc), though there was reduction in proliferation in both in-treatment and 24 h preconditioned cells as compared to untreated control.

We assessed the viability of the cells treated directly with CM-MDA231 (in-treatment) or preconditioned with different concentrations of CM-MDA231 (1–25%) which was found at 95%. CV-MSCs treated with higher concentrations of CM-MDA231 at 50 and 100% showed decreased viability at > 90%. Based on the results obtained above, the exposure time of 24 and 72 h was chosen for preconditioning of CV-MSCs with 25% of CM-MDA231 to study their functional outcome.

### CM-MDA231 Effects Adhesion and Proliferation of CV-MSCs

**FIGURE 2:**
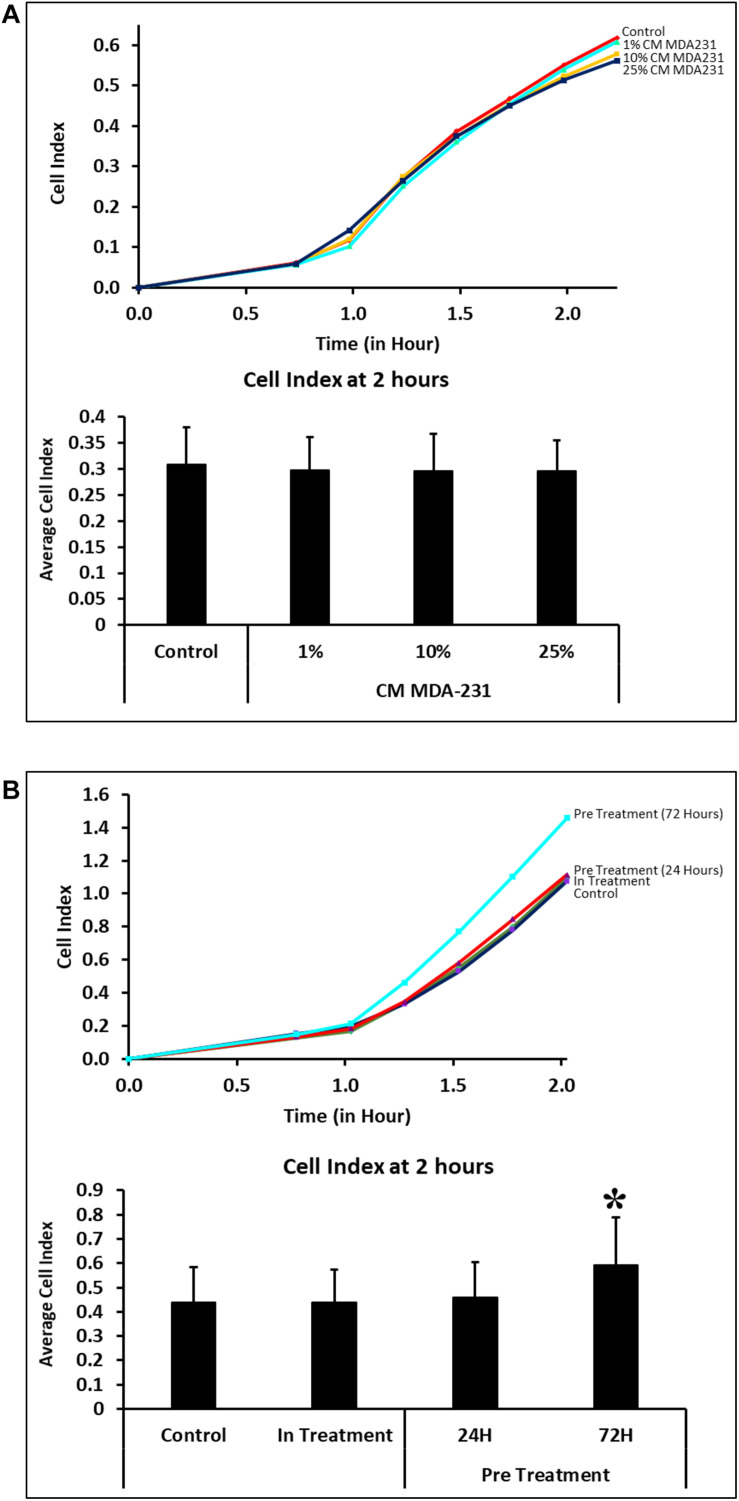
Adhesion of CV-MSCs in response to CM-MDA231 (in-treatment) and after preconditioning. Cellular adhesion data as observed with the xCELLigence system indicate that in response to 1–25% CMMDA-231 (under in-treatment conditions), CV-MSCs adhesion did not change significantly (*P* > 0.05) at 2 h as compared to untreated control **(A)**. However, prior preconditioning for 72 and subsequent adhesion analysis showed a significant increase in adhesion as compared to 24 h precondition, in-treatment and untreated control **(B)**. Each experiment was repeated three times with cells isolated from five independent placentae. Bars represent standard errors *P < 0.05.

Adhesion of CV-MSCs did not change in 2 h in the presence of various concentrations of CM-MDA231 (1–25%) in comparison to untreated control cells (Figure 2A). However, adhesion of cells preconditioned with 25% CM-MDA231 for 72 h increased significantly (*P* < 0.05), after 2 h of seeding in the xCELLigience system plates and as compared to cells preconditioned for 24 h only, or to in-treatment and untreated control (Figure 2B).

**FIGURE 3:**
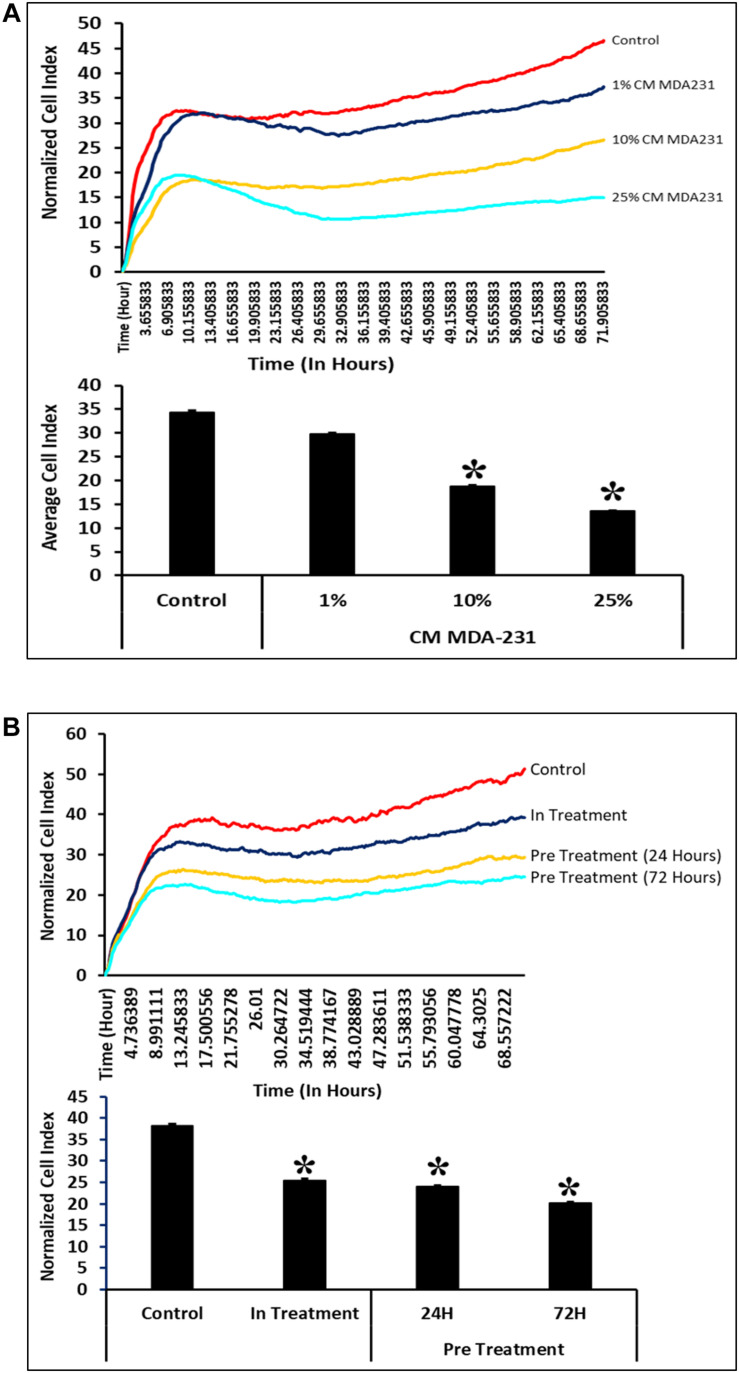
Comparison of functional outcome of CV-MSCs before and after CM-MDA231 preconditioning. Proliferation assessment by xCELLigence RTCA system after cells were treated with 1–25% of CM-MDA231 for 72 h (In-treatment) while performing the assay. CV-MSC proliferation reduced significantly at 10 and 25% treatment conditions as compared to 1% and untreated control **(A)**. Significantly, reduced proliferation was observed in CV-MSCs preconditioned for 24 and 72 h as well in the in-treatment conditions (at 25% CM-MDA231 concentration, while performing the assay) as compared to untreated control **(B)**. Experiments were performed in triplicate using cells at passage 3 prepared from five individual placentae. Bars represent standard errors *P ≤ 0.05).

CV-MSCs show significantly reduced (*P* < 0.05) proliferation in the presence of 10 and 25% of CM-MDA231. However, at 1% concentration of CM-MDA231, the cells did not show any significant change in proliferation, as compared to untreated control (Figure 3A). Exhibiting similar results, proliferation of CV-MSCs reduced significantly (*P* < 0.05) in in-treatment experimental group, and in the cells preconditioned for 24 and 72 h in proliferation assay using the xCELLigience RTCA system (Figure 3B).

### CM-MDA231 Effects Migratory Potential of CV-MSCs

**FIGURE 4:**
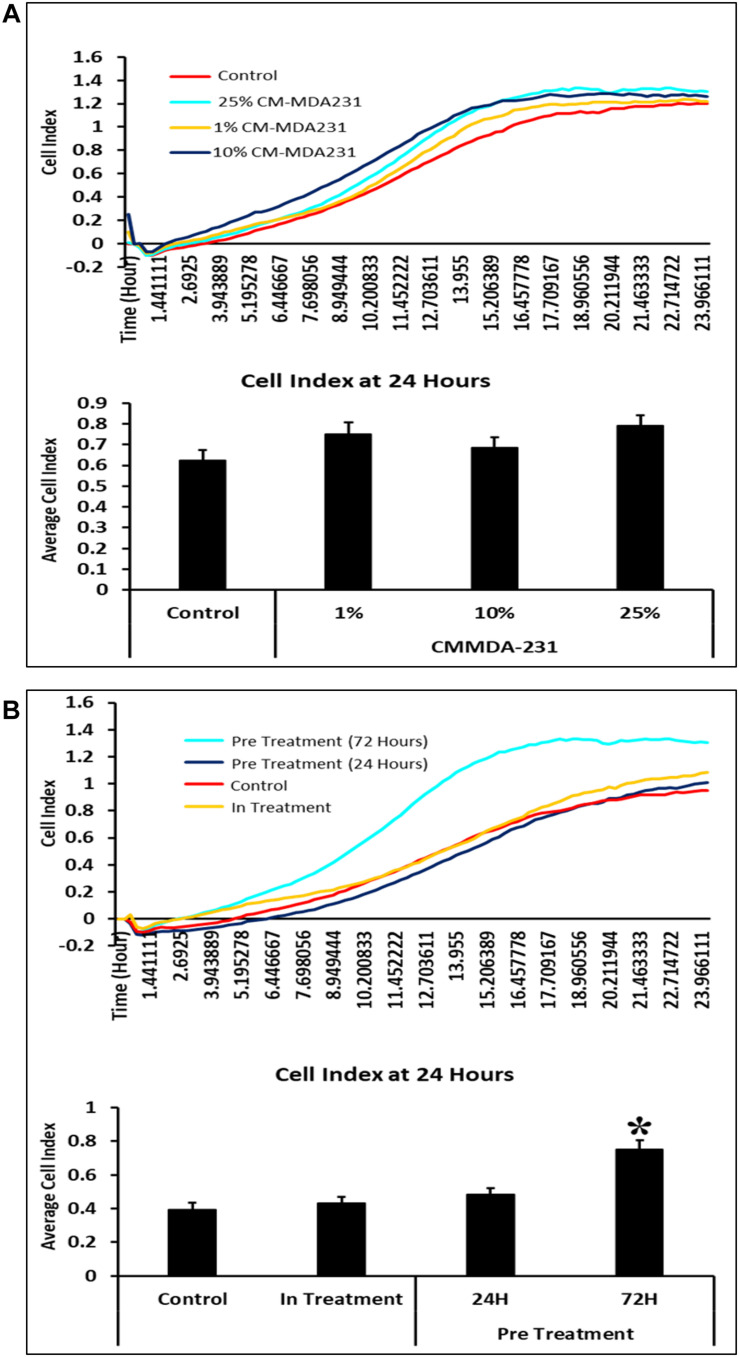
Comparison of functional outcome of CV-MSCs before and after CM-MDA231 preconditioning. Migration analysis assessment by xCELLigence system after cells were treated with 1–25% of CM-MDA231 for 24 h (In-treatment) while performing the assay. As shown, CV-MSC migration at any of the treatment conditions (1–25%), did not change as compared to untreated control **(A)**. However, significant increase in migration was observed in CV-MSCs preconditioned for 72 h as compared to cells preconditioned for 24 h, as well in the in-treatment conditions (at 25% CM-MDA231 concentration, while performing the assay) and untreated control cells **(B)**. Experiments were performed in triplicate using cells at passage 3 prepared from five individual placentae. Bars represent standard errors *P ≤ 0.05).

In response to 1–25% in-treatment of CM-MDA231, CV-MSC migration did not change as compared to the untreated control, in the xCELLigence RTCA system (Figure 4A). Preconditioning of CV-MSCs with 25% CM-MDA231 for 72 h but not for 24 h, significantly increased (*P* < 0.05) the migration rate of CV-MSCs at 24 h, as compared to in-treatment and untreated CV-MSCs controls (Figure 4B).

**FIGURE 5:**
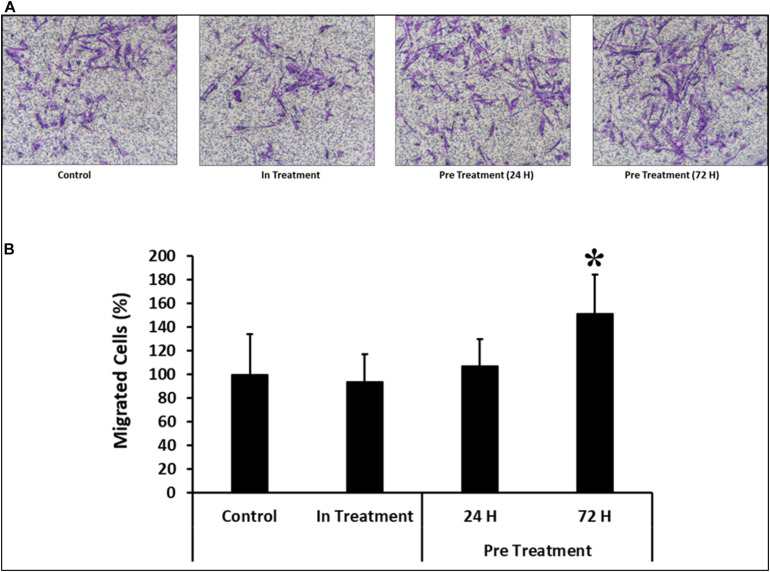
Comparison of functional outcome of CV-MSCs before and after CM-MDA231 preconditioning. Migration of CV-MSCs before and after preconditioning along-with in-treatment was further evaluated by transwell assay. CV-MSCs preconditioned with CM-MDA231 for 24 h, migrated at a significantly faster rate through the 8 μM pore of a transwell filter as compared to the cells treated for 24 h treatment, in-treatment and untreated control. **(A)** Shows the photomicrograph of the migrated cells under various treatment conditions. Five different fields were counted and the percentage of total migrated cells are presented in a bar graph **(B)**. Cells isolated from five individual placentae were used in these experiments, which were repeated three times. Bars represent standard errors *P ≤ 0.05).

Similar results were observed in the transwell assay, where preconditioned cells were made to pass through an insert with a pore size of 8 μM. As compared to the cells preconditioned for 24 h before the assay or in-treatment and untreated controls, the percentage of migrated cells was significantly higher (*P* < 0.05) in CV-MSCs preconditioned for 72 h prior to the assay (Figures 5A,B).

### Invasion of CV-MSCs in Response to CM-MDA231

**FIGURE 6:**
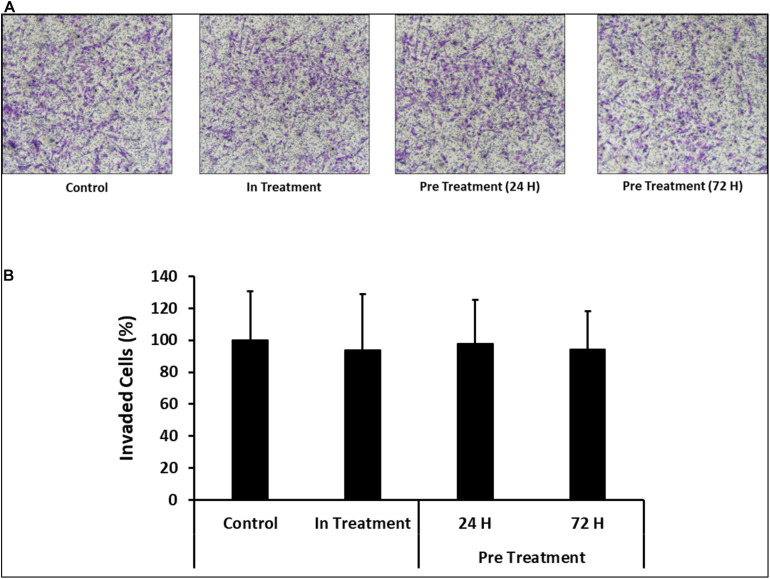
Comparison of functional outcome of CV-MSCs before and after CM-MDA231 preconditioning. Invasion of CV-MSCs before and after preconditioning, was examined by adding the cells to a transwell insert with a pore size of 8 μM, and covered with a layer of matrigel, as described in materials and methods section. After 24 h of culture, no significant change was observed in the invasion of cells preconditioned for 24 and 72 h as compared to in-treatment conditions and untreated control experiment groups. **(A)** Shows the photomicrograph of the migrated cells under various treatment conditions. Five different fields were counted and the percentage of total migrated cells are plotted in a bar graph **(B)**. Each experiment was performed three times and repeated with the cells isolated from five different placentae. Bars represent standard errors.

In order to assess the effect of CM-MDA231 on other cellular functions, CV-MSC invasion was examined using the transwell assay, where the inserts were coated with matrigel. The infiltration of matrigel by CV-MSCs define their invasion potential. After staining, the invaded cells were counted, and as compared to in-treatment group or untreated control, the invasion of CV-MSCs preconditioned with 25% CM-MDA231 for 24 and 72 h did not change significantly (*P* > 0.05) (Figures 6A,B), indicating that preconditioning with CM-MDA231 did not alter that invasive potential of CV-MSCs.

### Differential Expression of Functionally Relevant Effector Molecules

In order to decipher the effectors responsible for increased functionalities after preconditioning of CV-MSCs, the expression of a variety of effector molecules were studied by RT-PCR analysis, as well as by flow cytometry. We used a “RT^2^ Profiler Kit” for the expression evaluation of human cytokines and chemokines. As shown in Table 1 and Supplementary Tables 1–5, after 72 h of preconditioning a variety of effector molecules responsible for various cellular functions, including cellular proliferation, adhesion, apoptosis, inflammation and migration were found to be modulated many folds compared to in-treatment conditions or the untreated control. In addition, we studied various molecules, such as VCAM, ICAM1, PECAM, E-Cadherin, Integrin-2B, Integrin-α5, Integrin-M, and EpCAM, which play important roles in the cellular adhesion to their membrane, using flow cytometry. Expression of these molecules were recorded as median fluorescence intensity (MFI) units.

**TABLE 1:** Modulation of gene expression in CV-MSCs after preconditioning with CM-MDA231.

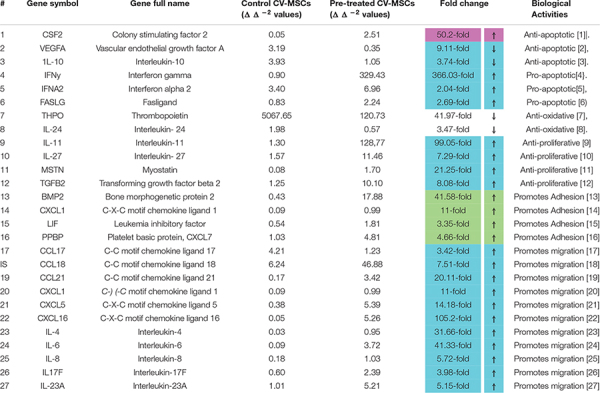

**Differential gene expression observed in CV-MSCs after preconditioning for 72 h with CM-MDA231, and normalized with untreated controls. RT-PCR was performed using RT^2^ profiler kit (Qiagen), as detailed in materials and methods. Three independent experiments were performed and data is expressed as fold change increase calculated from the ΔΔ^–^*^2^ values.*

**FIGURE 7:**
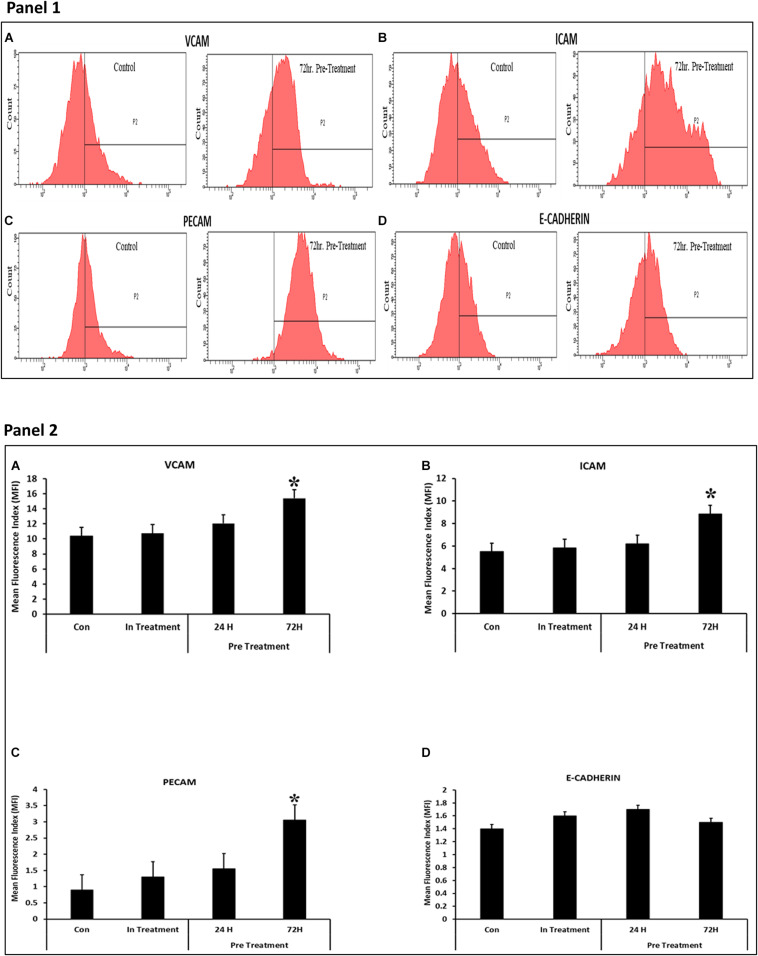
Effect of CM-MDA231 on CV-MSCs expression of Adhesion molecules. Flow cytometry analysis of the adhesion molecules performed on preconditioned, in-treatment, and untreated CV-MSCs showed that there was significant increase in the expression levels for VCAM, ICAM1 and PECAM, after preconditioning them for 72 h **(A–C)**, but no changes in the expression levels were observed in cells preconditioned for 24 h, in-treatment and untreated controls. No significant changes in expression levels was observed for E-Cadherin **(D)** between the preconditioned, in-treatment and untreated controls (Panel 1). The data obtained by FACS from five independent experiments was quantified. The average is presented as bar diagrams in panel 2. VCAM is represented as **(A)**, ICAM1 as **(B)**, PECAM as **(C)** and E-Cadherin as **(D)** in panel 2, respectively. Bars represent standard errors *P ≤ 0.05.

A number of molecules including VCAM, ICAM1, and PECAM were found to be differentially expressed in CV-MSCs after 72 h of preconditioning with CM-MDA231 (Figures 7A–D; Panels 1, 2). However, no change in expression was found in other molecules studied, including the E-Cadherin.

### Modulatory Effects of Preconditioning on Cell Cycle Regulation

**FIGURE 8:**
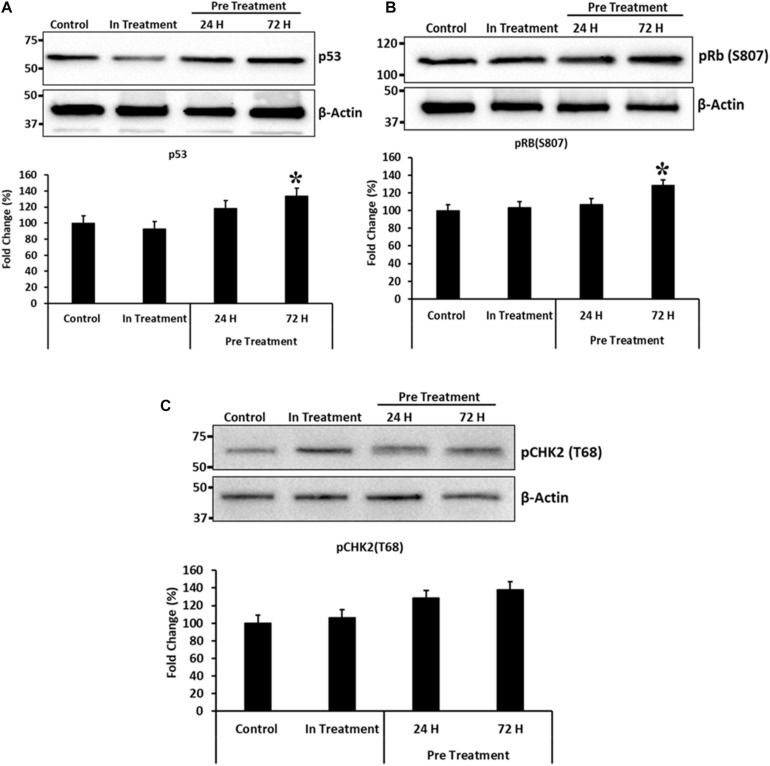
Immunoblotting for selected proteins involved in cell cycle regulation. Immunoblotting was performed on preconditioned, in-treatment, and untreated control CV-MSCs. Expression levels of p53 and phosphorylation of Rb(S807) proteins **(A,B)**, increased significantly at 72 h of preconditioning, as compared to the CV-MSCs preconditioned for 24 h, in-treatment and untreated controls. Although there was an increase in phosphorylation levels for CHK2(T68) protein **(C)** at 24 and 72-h post preconditioning as compared to in-treatment and untreated control, yet the levels were not statistically significant. Experiments were conducted in triplicate using cells prepared from five placentae. Bars represent standard errors and (*) represent the significance of the results (*P* ≤ 0.05).

To, further study the effects of CM-MDA231 preconditioning on cellular functions of CV-MSCs, and because preconditioning has resulted in significant decrease in their proliferative potential, we examined modulation of the proteins involved in cell cycle arrest, including the p53, Retinoblastoma (Rb) and Checkpoint kinase-2 (Chk2). As shown in Figure 8. CM-MDA231 treatment significantly enhanced the expression levels of p53 and also increased phosphorylation of Rb and Chk2. This increase in total levels as well as in phosphorylation was significant for the CV-MSCs preconditioned with CM-MDA231 for 72 h as compared to 24 h treatment (in-treatment) and untreated controls.

## Discussion

CV-MSCs isolated from the chorionic villi of human placentae express and secrete several important factors which have ability to modify the functional activities of their target cells (Abumaree et al., 2013b). They are multipotent stromal cells which undergo differentiation to other cell lineages and have immunosuppressive properties (Al Jumah and Abumaree, 2012; Abumaree et al., 2013a,b). We have reported earlier that CV-MSCs not only function normally in harsh oxidative stress environment induced by high levels of H_2_O_2_ and glucose (Al Jumah and Abumaree, 2012; Abumaree et al., 2013a; Basmaeil et al., 2020), but also protect endothelial cells from their detrimental effects (Abumaree et al., 2017; Basmaeil et al., 2018). In addition, we have also demonstrated that CV-MSCs induce anti-tumor properties of NK cells (Abumaree et al., 2013b). These distinct properties of CV-MSCs make them an attractive source for allogeneic transplantation to treat inflammatory diseases such as cancer, where the microenvironment contains high levels of oxidative stress and inflammatory mediators.

We extended our studies to examine if CV-MSCs are suitable for the treatment of inflammatory diseases such as cancer, and to investigate if they withstand the hostile environment like that of a tumor. Tumor microenvironment includes high levels of oxidative stress and inflammatory molecules (i.e., cytokines and chemokines) produced by tumor, tumor associated fibroblasts and immune cells (Uchibori et al., 2009; Arnold et al., 2015). This harsh environment proves an impediment for the successful outcome of otherwise beneficial MSCs, as it affects their phenotypic and functional abilities (Alphonso and Alahari, 2009; Burr et al., 2013; English, 2013; Yang et al., 2013; Guan, 2015; Lee et al., 2015; Lee and Hong, 2017). For successful transplantation and better outcome from CV-MSCs in inflammatory diseases, we investigated the functionalities of CV-MSCs while exposing them to a medium mimicking the cancer microenvironment (CM-MDA231), *in vitro*. In addition, the phenotypic and genotypic modulations were evaluated after preconditioning of CV-MSCs with CM-MDA231.

In order to evaluate the functional consequences of CV-MSCs after CM-MDA231 treatment, it was necessary to determine an appropriate dose of CM-MDA231 to have a measurable outcome in a given time on the CV-MSCs. This spatial and temporal measurements were performed simultaneously while treating the cells directly and measuring the functional outcome (in-treatment), or preconditioning the cells for a specific period of time with a specific doze of CM-MDA231 (preconditioning or pre-treatment), and then measuring its effect.

Treatment of CV-MSCs with different concentrations of conditioned media did not change the viability of CV-MSCs even up to the high concentration (up to 100%) of CM-MDA231. Expression of high level of anti-apoptotic gene, such as CSF2 (Loureiro et al., 2011; Table 1) and low level of pro-apoptotic genes (i.e., IFNα2, IFNγ, and FASLG (Croker et al., 2011; Kotredes and Gamero, 2013; Badie et al., 2000), by preconditioned CV-MSCs, may explain their survival potential in the hazardous environment of CM-MDA231 (Table 1 and Supplementary Tables 1, 2). We confirmed the stemness of preconditioned CV-MSCs by analyzing the expression of stem cell markers and absence of hematopoietic stem cell markers before performing any functional assay. Treated cells retained all the properties of the parent cells. Preconditioned CV-MSCs were positive for all the stem cell markers including the HLA-ABC and were negative for all the hematopoietic markers, the endothelial cell markers and costimulatory molecules. Expression or loss of these molecules did not change throughout the culture. Morphologically, these cells resembled the untreated controls, and formed a homogenous monolayer of adherent cells on plastic.

Conditioned medium obtained from various cancer cells grown in culture (*in vitro*) contain various molecules involved in inflammatory and apoptotic activities. They include mostly interleukins, such as IL-1β, IL-6, IL-8, cytokines, and MMPs (matrix metalloproteinase’s) (Bachmeier et al., 2001; Freund et al., 2003; Tan et al., 2006; Thirumangalakudi et al., 2007; Lieblein et al., 2008; Oh et al., 2011; Escobar et al., 2015; Shen et al., 2017). The ability of CV-MSCs to overcome the adverse effects of these molecules is in accordance to our previous study, which demonstrated the resistance of CV-MSCs to the lethal effects of H_2_O_2_ and glucose (Abumaree et al., 2017; Basmaeil et al., 2018). The ability of preconditioned CV-MSCs to counteract the detrimental effects of these molecules can be explained by their expression of genes with various anti-oxidant properties (THPO and IL-24) (Chan et al., 2015; Wang et al., 2016) and downregulation of anti-apoptotic genes (VEGFA and IL-10) (Gerber et al., 1998; Zhou et al., 2001), as summarized in Table 1 and Supplementary Tables 1, 2.

The survival and retention of functional activities (except proliferation) of CV-MSCs in the inflammatory condition supports the concept that cancer microenvironment may not modulate their functional consequences in totality. Although, the in-treatment and preconditioned cells exhibit reduced proliferation rate (Figure 3), yet this effect may be due to temporary senescence or cell cycle arrest initiated in response to the stresses created by the CM-MDA231, which is a common phenomenon observed during cellular therapy against cancer (Lee and Lee, 2019). CV-MSCs proliferated under continuous effect of CM-MDA231 (Figure 3A) at lower percentage, but showed significantly reduced proliferation potential at higher percentage (10 and 25%; Figure 3A). However, preconditioning with CM-MDA231 for (24 and 72 h) reduced the proliferation of CV-MSCs significantly (Figure 3B). This reduction in proliferation of CV-MSCs exhibit their safety after transplantation, as it reduces the chances of teratoma or tumor formation. Similar phenotype was observed for human bone marrow-derived MSCs (BMMSCs) also; where it is reported that transplantation of BMMSCs is not associated with tumor formation (Wang et al., 2016). Therefore, CV-MSCs may be safely transplanted in patients with various inflammatory diseases, where stem cell therapy is an option. However, further investigation in needed to prove the hypothesis. The inhibitory effect of CM-MDA231 on proliferation of CV-MSCs could be attributed to the anti-proliferative molecules, secreted by MDA-231 into the conditioned medium, such as parathyroid hormone-related protein (PTHrP), plasminogen activator inhibitor type 1 (PAI-1) and IL-10 (Taga and Tosato, 1992; Freund et al., 2003; Morgan et al., 2004; Kouri et al., 2008; Zhang et al., 2018). Exposure of CV-MSCs to CM-MDA231 enhanced their expression of anti-proliferative genes such as, IL-11, IL-27, MSTN, and TGFβ2, which could be the basis of inhibition of proliferation of CV-MSCs (Hurteau et al., 1994; Taylor et al., 2001; Paiva et al., 2007; Xu et al., 2017; Table 1 and Supplementary Tables 1, 2). Increase in expression of p53 and phosphorylation of Retinoblastoma (Rb) and Checkpoint kinase-2 (Chk2) in preconditioned CV-MSCs, signifies that decrease in proliferation may be because of the cell cycle arrest (Figure 8). However, the exact mechanism underlying the anti-proliferative effects CM-MDA231 on CV-MSCs needs to be examined in future studies.

Continuous culture of CV-MSCs in CM-MDA231 did not alter their adhesion potential (Figure 2A) while preconditioning with CM-MDA231 enhanced their adhesive properties significantly at 72 h post preconditioning (Figure 2B). Increased adhesion of preconditioned CV-MSCs may be achieved via a mechanism that may involve numerous adhesive genes such as BMP2, CXCL1, LIF and PPBP, which were identified in RT-PCR of the RNA isolated from preconditioned cells (Shah et al., 1999; Schenk et al., 2002; Tapia et al., 2008; Roy et al., 2012; Table 1 and Supplementary Tables 1, 2). However, the exact role of each molecule needs to be investigated in future studies. In addition, the significant increase in adhesion molecules including, VCAM, ICAM1, and PECAM, but not in E-Cadherin levels as observed in FACS analysis justified the increase in adhesion potential of the preconditioned CV-MSCs preconditioned (Figure 7).

Our results showed that continuous treatment of CV-MSCs with a specific concentration of CM-MDA231 did not alter their migration potential (Figure 4A). However, preconditioning with CM-MDA231 enhanced their migratory properties (Figure 4B), as compared to in-treatment and untreated controls. Genomic analysis of preconditioned cells revealed upregulation of multiple chemokines and interleukins compared to the untreated control (Table 1 and Supplementary Tables 1, 2). Most of these effectors are responsible for cellular migration (Mori et al., 2000; Itoh et al., 2005; Stutte et al., 2010; Kuo et al., 2012; Hu et al., 2014; Ota et al., 2014; Xu et al., 2014; Mei et al., 2015; Mo et al., 2015; Razidlo et al., 2018; Liu et al., 2019), and their upregulation arguably proves the increase of migration of preconditioned CV-MSCs.

For successful stem cell transplantation, MSCs must invade and penetrate (engraftment) the endothelium or endothelial cells lining the walls of blood vessels in order to reach their cellular and tissue targets. Yet, aggressive invasion and long term retention may result in increased teratoma formation, which may be detrimental for the patient in long run (Sid-Otmane et al., 2020). In-treatment or preconditioning of CV-MSCs with CM-MDA231 did not change their invasive properties as shown in Figure 6. These data show the ability of CV-MSCs to perform important cellular functions without compromising the patient integrity, which is the hallmark of stem cell transplantation. Successful stem cell therapy is associated with functioning in diseased microenvironment under high levels of oxidative stress and inflammatory mediators without teratoma or tumor formation (Frijns and Kappelle, 2002; Veevers-Lowe et al., 2011).

In addition to modulation in expression of effector molecules such as chemokines, cytokines, growth factors and adhesion molecules, involved in various cellular functions, including proliferation, migration, invasion and adhesion, preconditioned CV-MSCs expressed high levels of a variety of anti-inflammatory molecules (Supplementary Table 3). The expression of such molecules validate their immunosuppressive properties, as has been reported earlier (Hartung, 1998; Hermann et al., 1998; Sanjabi et al., 2009; Alikhan et al., 2011; Li et al., 2014; Davis et al., 2018). This is in agreement with our previous studies where we have reported the immunosuppressive functional activities of CV-MSCs on immune cells, including monocytes/macrophages, dendritic cells and T-cells (Al Jumah and Abumaree, 2012; Abumaree et al., 2013a; Abomaray et al., 2015). Preconditioned CV-MSCs also expressed high levels of pro-inflammatory genes (Supplementary Table 4). Expression of pro-inflammatory molecules may be justified with the fact that preconditioning helps to prime CV-MSCs, and push them toward an anti-inflammatory and inflammatory phenotype, as previously reported. It has been shown that priming human MSCs by Toll-like receptors polarize them into inflammatory MSC 1 or an immunosuppressive MSC 2 phenotype (Mathy et al., 2000; Zlotnik and Yoshie, 2000; Gombert et al., 2005; De Plaen et al., 2006; Lue et al., 2007; Croft, 2009; Waterman et al., 2010; Veevers-Lowe et al., 2011; Cao et al., 2014; Fabre et al., 2016; Chan et al., 2017; Scheu et al., 2017).

Although CV-MSCs in both in-treatment as well as after preconditioning survived the toxic effect of CM-MDA231, yet they showed significantly reduced proliferation. In order to understand the rationale behind this phenotype, we asked if cell cycle checkpoints or apoptotic pathways were affected after preconditioning. Based on the evidence obtained from the genomic analysis, where modulation in the expression of a plethora of pro-apoptotic as well as pro-survival molecules were observed (Table 1 and Supplementary Tables 1, 2), the expression levels of p53 and phosphorylation profiles of CHK2 and Rb were evaluated for validation purposes. Results indicated that there was a significant increase in the total expression p53 and phosphorylation of Chk2 and Rb in CV-MSCs, after preconditioning (Figure 8), indicating that decreased proliferation of CV-MSCs may be mediated via all or one of these pathways. p53 is tumor suppressor, and functions also as a transcription factor. Under various stresses, it activates anti-proliferative pathways by activating or repressing key effector genes (Zilfou and Lowe, 2009). Similarly, Chk2 is activated by phosphorylation in cells under stress, and induces multiple cellular response, which include cell cycle checkpoint activation, induction of senescence and/or apoptosis (Zannini et al., 2014). Among all known tumor suppressor genes, the retinoblastoma (RB) is known as a master regulator of the cell cycle. It is involved in various cellular functions, including DNA repair, telomere maintenance, chromosome condensation etc. (Vélez-Cruz and Johnson, 2017).

The findings of this study provide a new evidence in support of CV-MSCs to be used safely in inflammatory diseases, such as cancer. The survival and normal functioning of CV-MSCs in a toxic medium mimicking cancer microenvironment will open new therapeutic avenues where CV-MSCs can be safely transplanted in similar disease setups. Since, their immunomodulatory properties have already been established, this study provide an additional insight in that direction. The finding of this study will reduce the bias toward placental mesenchymal stem cells, and provide a new approach of selecting the type of MSCs that suits the inflammatory disease under investigation.

## Conclusion

This study investigates the effects of medium mimicking cancer microenvironment on the survival and other functional activities of CV-MSCs. Conditioned media from cancer cells inhibit CV-MSCs proliferation indicating that transplantation of CV-MSCs may not be associated with tumor/teratoma formation, thus demonstrating the biosafety of CV-MSCs for their therapeutic uses. CV-MSCs performed normally for other functional activities, including adhesion, migration and invasion. In addition, preconditioned CV-MSCs showed differential expression of pro- and anti-inflammatory molecules, enhancing their chances of survival in hostile environment. These data demonstrate the suitability of using CV-MSCs in treating inflammatory disease like cancer. However, a comprehensive future study must be executed: (1) to confirm the mechanism underlying the normal functioning of CV-MSCs in unfriendly atmosphere and, (2) the retention of stem cell properties to deliver their therapeutic pay loads successfully.

## Data Availability Statement

The original contributions presented in the study are included in the article/Supplementary Material, further inquiries can be directed to the corresponding author/s.

## Ethics Statement

The studies involving human participants were reviewed and approved by the King Abdullah International Medical Research Center. The patients/participants provided their written informed consent to participate in this study.

## Author Contributions

TK and MA proposed the study. TK designed and supervised the experiments. AA and YB performed the experiments. TK and YB analyzed the data and interpreted the results. TK wrote the manuscript. YB, AA, and TK reviewed the manuscript before submitting for publication. All authors contributed to the article and approved the submitted version.

## Conflict of Interest

The authors declare that the research was conducted in the absence of any commercial or financial relationships that could be construed as a potential conflict of interest.
