# Supplementary material for: Conditions Mimicking the Cancer Microenvironment Modulate the Functional Outcome of Human Chorionic Villus Mesenchymal Stem/Stromal Cells in vitro
Source: Front Cell Dev Biol. 2021 Jun 21;9:650125. doi: 10.3389/fcell.2021.650125 (PMC8255990; doi:10.3389/fcell.2021.650125)
Supplement: Supplementary file 1 [file Data_Sheet_1.docx]

**Supplementary Table 1:** RT-PCR analysis of genes modulated in CV-MSCs after CM-MDA231 treatment. CM-MDA231 modulated CV-MSC expression of anti-apoptotic, pro-apoptotic, anti-oxidant, proliferative, anti-proliferative and adhesive genes.

| **#** | **Gene Symbol** | **Gene Full Name** | **Control CV-MSCs**  (ΔΔ^-2^ values) | **Pre-Treated CV-MSCs**  (ΔΔ^-2^ values) | **Fold Change** | | **Biological Activities** |
| --- | --- | --- | --- | --- | --- | --- | --- |
| 1 | CSF2 | Colony stimulating factor 2 | 0.05 | 2.51 | 50.2 Fold | 🡹 | Anti-apoptotic |
| 2 | VEGFA | Vascular endothelial growth factor A | 3.19 | 0.35 | 9.11 Fold | 🡻 | Anti-apoptotic |
| 3 | IL-10 | Interleukin- 10 | 3.93 | 1.05 | 3.74 Fold | 🡻 | Anti-apoptotic |
| 4 | IFNγ | Interferon gamma | 329.43 | 0.90 | 366.03 Fold | 🡻 | Pro-apoptotic |
| 5 | IFNA2 | Interferon alpha 2 | 6.96 | 3.40 | 2.04 Fold | 🡻 | Pro-apoptotic |
| 6 | FASLG | Fas ligand | 2.24 | 0.83 | 2.69 Fold | 🡻 | Pro-apoptotic |
| 7 | THPO | Thrombopoietin | 5067.65 | 120.73 | 41.97 Fold | 🡻 | Anti-oxidative |
| 8 | IL-24 | Interleukin- 24 | 1.98 | 0.57 | 3.47 Fold | 🡻 | Anti-oxidative |
| 9 | IL-11 | Interleukin- 11 | 1.30 | 128.77 | 99.05 Fold | 🡹 | Anti-proliferative |
| 10 | IL-27 | Interleukin- 27 | 1.57 | 11.46 | 7.29 Fold | 🡹 | Anti-proliferative |
| 11 | MSTN | Myostatin | 0.08 | 1.70 | 21.25 Fold | 🡹 | Anti-proliferative |
| 12 | TGFB2 | Transforming growth factor beta 2 | 1.25 | 10.10 | 8.08 Fold | 🡹 | Anti-proliferative |
| 13 | BMP2 | Bone morphogenetic protein 2 | 0.43 | 17.88 | 41.58 Fold | 🡹 | Promotes adhesion |
| 14 | CXCL1 | C-X-C motif chemokine ligand 1 | 0.09 | 0.99 | 11 Fold | 🡹 | Promotes adhesion |
| 15 | LIF | Leukemia inhibitory factor | 0.54 | 1.81 | 3.35 Fold | 🡹 | Promotes adhesion |
| 16 | PPBP | Platelet basic protein, CXCL7 | 1.03 | 4.81 | 4.66 Fold | 🡹 | Promotes adhesion |

**Supplementary Table 2:** RT-PCR analysis of genes modulated in CV-MSCs after CM-MDA231 treatment. CM-MDA231 modulated CV-MSC expression of genes associated with cellular migration.

| **#** | **Gene Symbol** | **Gene Full Name** | **Control CV-MSCs**  (ΔΔ^-2^ values) | **Pre-Treated CV-MSCs**  (ΔΔ^-2^ values) | **Fold Change** | | **Biological Activities** |
| --- | --- | --- | --- | --- | --- | --- | --- |
| 1 | CCL17 | C-C motif chemokine ligand 17 | 4.21 | 1.23 | 3.42 Fold | 🡹 | Promotes migration |
| 2 | CCL18 | C-C motif chemokine ligand 18 | 6.24 | 46.88 | 7.51 Fold | 🡹 | Promotes migration |
| 3 | CCL21 | C-C motif chemokine ligand 21 | 0.17 | 3.42 | 20.11 Fold | 🡹 | Promotes migration |
| 4 | CXCL1 | C-X-C motif chemokine ligand 1 | 0.09 | 0.99 | 11 Fold | 🡹 | Promotes migration |
| 5 | CXCL5 | C-X-C motif chemokine ligand 5 | 0.38 | 5.39 | 14.18 Fold | 🡹 | Promotes migration |
| 6 | CXCL16 | C-X-C motif chemokine ligand 16 | 0.05 | 5.26 | 105.2 Fold | 🡹 | Promotes migration |
| 7 | IL-4 | Interleukin- 4 | 0.03 | 0.95 | 31.66 Fold | 🡹 | Promotes migration |
| 8 | IL-6 | Interleukin- 6 | 0.09 | 3.72 | 41.33 Fold | 🡹 | Promotes migration |
| 9 | IL-8 | Interleukin- 8 | 0.18 | 1.03 | 5.72 Fold | 🡹 | Promotes migration |
| 10 | IL17F | Interleukin- 17F | 0.60 | 2.39 | 3.98 Fold | 🡹 | Promotes migration |
| 11 | IL-23A | Interleukin- 23A | 1.01 | 5.21 | 5.15 Fold | 🡹 | Promotes migration |

**Supplementary Table 3:** RT-PCR analysis of genes modulated in CV-MSCs after CM-MDA231 treatment. CM-MDA231 modulated CV-MSC expression of genes with inflammatory properties.

| **#** | **Gene Symbol** | **Gene Full Name** | **Control CV-MSCs**  (ΔΔ^-2^ values) | **Pre-Treated CV-MSCs**  (ΔΔ^-2^ values) | **Fold Change** | | **Biological Activities** |
| --- | --- | --- | --- | --- | --- | --- | --- |
| 1 | IL-8 | Interleukin- 8 | 0.18 | 1.03 | 5.72 Fold | 🡹 | Promotes inflammation |
| 2 | IL-16 | Interleukin- 16 | 0.12 | 2.97 | 24.75 Fold | 🡹 | Promotes inflammation |
| 3 | IL17F | Interleukin- 17F | 0.60 | 2.39 | 3.98 Fold | 🡹 | Promotes inflammation |
| 4 | IL-23A | Interleukin- 23A | 1.01 | 5.21 | 5.15 Fold | 🡹 | Promotes inflammation |
| 5 | CCL1 | Chemokine (C-C motif) ligand 1 | 0.094 | 5.60 | 59.57 Fold | 🡹 | Promotes Inflammation |
| 6 | CCL7 | Chemokine (C-C motif) ligand 7 | 0.02 | 1.10 | 55 Fold | 🡹 | Promotes inflammation |
| 7 | CCL22 | Chemokine (C-C motif) ligand 22 | 0.06 | 1.41 | 23.5 Fold | 🡹 | Promotes inflammation |
| 8 | CXCL1 | C-X-C motif chemokine ligand 1 | 0.09 | 0.99 | 11 Fold | 🡹 | Promotes inflammation |
| 9 | CXCL2 | C-X-C motif chemokine ligand 2 | 121.63 | 37457.57 | 307.95 Fold | 🡹 | Promotes inflammation |
| 10 | MIF | Macrophage migration inhibitory factor | 0.16 | 20.95 | 130.93 Fold | 🡹 | Promotes inflammation |
| 11 | TNFSF11 | TNF superfamily member 11 | 0.35 | 1.36 | 3.88 Fold | 🡹 | Promotes inflammation |
| 12 | TNFSF13B | TNF superfamily member 13B | 0.36 | 1.12 | 3.11 Fold | 🡹 | Promotes inflammation |

**Supplementary Table 4:** RT-PCR analysis of genes modulated in CV-MSCs after CM-MDA231 treatment. CM-MDA231 modulated CV-MSC expression of genes with anti-inflammatory properties.

| **#** | **Gene Symbol** | **Gene Full Name** | **Control CV-MSCs**  (ΔΔ^-2^ values) | **Pre-Treated CV-MSCs**  (ΔΔ^-2^ values) | **Fold Change** | | **Biological Activities** |
| --- | --- | --- | --- | --- | --- | --- | --- |
| 1 | IL-11 | Interleukin- 11 | 1.30 | 128.77 | 99.05 Fold | 🡹 | Anti-inflammatory |
| 2 | CSF1 | Colony-stimulating factor 1 | 0.32 | 3.08 | 9.62 Fold | 🡹 | Anti-inflammatory |
| 3 | CSF3 | Colony-stimulating factor 3 | 0.05 | 1.93 | 38.6 Fold | 🡹 | Anti-inflammatory |
| 4 | BMP4 | Bone morphogenetic protein-4 | 0.40 | 1.70 | 4.25 Fold | 🡹 | Anti-inflammatory |
| 5 | LIF | Leukemia inhibitory factor | 0.54 | 1.81 | 3.35 Fold | 🡹 | Anti-inflammatory |
| 6 | TGFB2 | Transforming growth factor beta 2 | 1.25 | 10.10 | 8.08 Fold | 🡹 | Anti-inflammatory |

**Supplementary Table 5:** RT-PCR analysis of genes modulated in CV-MSCs after CM-MDA231 treatment. CM-MDA231 modulated CV-MSC expression of other genes with varied functions. However, the fold change increase was not statistically significant.

| **#** | **Gene Symbol** | **Control CV-MSCs**  (ΔΔ^-2^ values) | **Pre-Treated CV-MSCs**  (ΔΔ^-2^ values) | **Fold Change** |
| --- | --- | --- | --- | --- |
| 1 | IL-1A | 0.07 | 0.09 | **Fold change is not statistically significant**  **P> 0.05** |
| 2 | IL-1B | 1.07 | 0.85 |  |
| 3 | IL-1RN | 0.09 | 0.24 |  |
| 4 | IL-5 | 0.08 | 0.04 |  |
| 5 | IL-7 | 0.91 | 1.17 |  |
| 6 | IL-12A | 0.0001 | 0.001 |  |
| 7 | IL-18 | 0.001 | 0.006 |  |
| 8 | CCL2 | 0.05 | 0.24 |  |
| 9 | CCL3 | 0.001 | 0.04 |  |
| 10 | CCL5 | 0.10 | 0.57 |  |
| 11 | CCL8 | 0.05 | 0.11 |  |
| 12 | CCL19 | 0.18 | 0.78 |  |
| 13 | CCL24 | 0.02 | 0.10 |  |
| 14 | CXCL10 | 0.001 | 0.0005 |  |
| 15 | CXCL11 | 0.73 | 1.12 |  |
| 16 | CXCL12 | 0.0001 | 0.001 |  |
| 17 | CX3CL1 | 0.32 | 0.44 |  |
| 18 | XCL1 | 1.99 | 2.31 |  |
| 19 | BMP6 | 0.038 | 0.006 |  |
| 20 | BMP7 | 0.20 | 0.003 |  |
| 21 | LTA | 0.92 | 0.81 |  |
| 22 | LTB | 0.02 | 0.06 |  |
| 23 | NODAL | 0.04 | 0.64 |  |
| 24 | OSM | 3.00 | 2.23 |  |
| 25 | SPP1 | 0.06 | 0.70 |  |
| 26 | TNFRSF11B | 0.13 | 0.13 |  |
| 27 | TNFSF10 | 0.0001 | 0.01 |  |
| 28 | TNF | 4.81 | 2.67 |  |
| 29 | GBI | 0.05 | 0.82 |  |
| 30 | CD40LG | 1.32 | 1.36 |  |
| 31 | C5 | 0.10 | 0.28 |  |
| 32 | ADIPOQ | 4.23 | 2.13 |  |
| 33 | CNTF | 0.03 | 0.02 |  |
